# Comparative metabolic state of microflora on the surface of the anode electrode in a microbial fuel cell operated at different pH conditions

**DOI:** 10.1186/s13568-016-0299-4

**Published:** 2016-12-20

**Authors:** Daisuke Sasaki, Kengo Sasaki, Yota Tsuge, Akihiko Kondo

**Affiliations:** 1 Graduate School of Science, Technology and Innovation, Kobe University, 1-1 Rokkodaicho, Nada-ku, Kobe, Hyogo 657-8501 Japan; 2 Institute for Frontier Science Initiative, Kanazawa University, Kakuma-machi, Kanazawa, Ishikawa 920-1192 Japan; 3 Graduate School of Engineering, Kobe University, 1-1 Rokkodai-cho, Nada-ku, Kobe, Hyogo 657-8501 Japan; 4 RIKEN Center for Sustainable Resource Science, 1-7-22 Suehiro-cho, Tsurumi-ku, Yokohama, Kanagawa 230-0045 Japan

**Keywords:** Microbial fuel cell, Microflora, Intracellular metabolomic analysis, TCA cycle, ATP generation

## Abstract

The metabolic state of microflora (mixed microbial cultures) in microbial fuel cells (MFCs) is currently unclear. Metabolomic analyses were conducted of microflora growing on the anodic electrodes of MFCs operated at pH 7.0, 5.5, or 4.0 and utilizing starch as the major carbon substrate. A much higher current was produced at pH 7.0 than at pH 5.5 and 4.0, correlating with an increased population ratio of *Geobacter* species to the total bacteria growing on the electrode. Most intracellular metabolites related to the tricarboxylic acid (TCA) cycle were present at a higher level at pH 7.0 than at pH 5.5 and 4.0, and the levels of metabolites correlated well with the obtained current densities. A high intracellular adenosine triphosphate (ATP)/adenosine diphosphate (ADP) ratio at pH 7.0, compared to at pH 5.5 and 4.0, likewise supported current production. Overall, the metabolomic analyses demonstrated that activation of the TCA cycle and increased ATP generation are critical parameters for electricity generation by microflora.

**Electronic supplementary material:**

The online version of this article (doi:10.1186/s13568-016-0299-4) contains supplementary material, which is available to authorized users.

## Introduction

Microbial fuel cells (MFCs) are being investigated for the simultaneous treatment of organic and inorganic materials such as wastewater and for generating electricity by using mixed cultures of microflora as the catalyst (Logan et al. 2006; Lovley 2008). Electrons generated by oxidation of the reduced substrate at the anode flow to the cathode through the circuit, resulting in the reduction of oxygen (Logan 2009). In most cases, MFCs employ microflora rather than a pure culture because mixed cultures can utilize complex organic materials as substrates and enhance anodophilic electron transfer due to interspecies connections (Logan et al. 2006). The effects of system architecture and operational parameters on electricity generation in MFCs have been intensively studied (Logan et al. 2006). Air-cathode MFCs, in which the cathode is in direct contact with the air, are useful because there is no need for aeration or chemical catholytes, there is high electrical output, and they are suitable for scale-up (Shimoyama et al. 2008). Electricity generation can be modulated by pH, which is one important parameter (He et al. 2008); however, although the pH range for adequate current generation is relatively broad (between pH 7 and 10), pH conditions close to neutral are usually used in MFCs to support the growth of anodic bacteria in air-cathode systems (He et al. 2008). To date, the conditions supporting efficient bacterial growth and current generation have been investigated, but less effort has been devoted to investigating the intracellular metabolic states of anode-respiring microflora in air-cathode MFCs.

*Geobacter sulfurreducens* is the primary microorganism used for current production and its electron transfer mechanism has been well studied (Lovley 2012). Recent advances in metabolomic analysis allow clarification of the intracellular metabolic states of microorganisms (Toya and Shimizu 2013) and this approach has shown that pure cultures of *G. sulfurreducens* activate the tricarboxylic acid (TCA) cycle and down-regulate gluconeogenesis under conditions compatible with electricity generation (Song et al. 2016). Metabolomic analysis can also be applied to microflora to understand the metabolic state of a bacterial community as a whole. In microflora used for methane fermentation, the Embden-Meyerhof (EM) pathway is inhibited and simultaneously the reductive branch of the TCA cycle is stimulated when the pH is decreased from 7.5 to 5.0, and methane fermentation is inhibited (Sasaki et al. 2014). This finding suggests that metabolomic analysis would be useful for evaluating the conditions for optimizing the performance of microflora in MFCs.

The aim of this study was to clarify the metabolic state of microflora on the surface of the anodic electrode in an air-cathode MFC operated at pH 7.0 to generate current. The metabolic states of microflora in comparable MFCs operated at pH 5.5 or 4.0 and producing a relatively low current were used as controls. Starch was the major carbon source in the substrate, simulating wastewater containing carbohydrate.

## Materials and methods

### MFC configuration

**Fig. 1:**
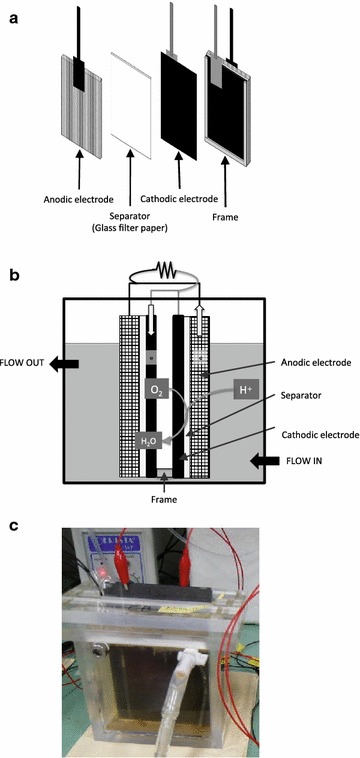
**a** Schematic diagram of anode and air cathode electrodes, **b** microbial fuel cell (MFC) and **c** photograph of the MFC used in this study

The MFC reactor had one cassette-electrode comprising an air cathode, a separator, and an anode, and this construction was mirrored on the other side of the air inlet interspace. This reactor represents a minor modification of a previously described reactor (Miyahara et al. 2013) (Fig. 1). Carbon paper (TGP-H-120; MICLAB, Kanagawa, Japan) coated with four 4-polytetrafluoroethylene (PTFE) layers and 1 mg cm^−2^ of a cathode catalyst [Pt-carbon (TEC10E70TPM); Tanaka Kikinzoku Kogyo, Tokyo, Japan] was used as the air cathode, and graphite felt (F-203G; Sohgoh Carbon, Yokohama, Japan) and a glass filter (GF/A; GE Healthcare, Little Chalfont, UK) were utilized for the anode and separator, respectively. The reactor had a total anode or cathode area of 144 cm^2^ in the one cassette-electrode and a total volume of 250 mL fermentation broth.

### Operation of the MFC

The MFC reactor was filled with synthetic wastewater (Guerrero et al. 2009) adjusted to pH 7.0, 5.5, or pH 4.0 using 20 mM phosphate buffer. The composition of the synthetic wastewater was as follows: starch (200 mg L^−1^), Bactopeptone (21 mg L^−1^), Bactoyeast extract (100 mg L^−1^), Tween-20 (20 mg L^−1^), urea (13 mg L^−1^), KH_2_PO_4_ (5.3 mg L^−1^), CaCl_2_·2H_2_O (22 mg L^−1^), MgSO_4_·7H_2_O (0.43 mg L^−1^), KCl (21 mg L^−1^), NaHCO_3_ (8.8 mg L^−1^) and 1 mL L^−1^ of a trace-element solution [Deutsche Sammlung von Mikroorganismen und Zellkulturen (DSMZ) medium 318; Braunschweig, Germany]. The MFCs were filled with either pH 7.0, 5.5, or 4.0 synthetic wastewater and duplicate experiments were conducted under each condition. MFC operation was initiated by inoculating with 20 mL activated sludge obtained from a sewage treatment plant operated by the Tokyo Metropolitan Government. The synthetic wastewater was provided at a flow rate of 250 mL day^−1^ (hydraulic-retention time of 24 h) at an initial external resistance (*R*_*ext*_) setting of 10 kΩ. Voltage (V) produced by the MFC was monitored and recorded using a data logger (NR-1000; Keyence, Osaka, Japan), and current (I) and power (P) were calculated from the voltage at the set *R*_*ext*_ using the equations I = V/*R*_*ext*_ and P = IV, respectively. The *R*_*ext*_ was dropped stepwise to 51 Ω as the voltage increased in the MFC operated at pH 7.0.

### Analysis of performances of the MFC

Chemical oxygen demand (COD) was measured with a COD reactor (DRB200, Hach, Loveland, CO, USA) using the dichromate method and COD removal efficiency (%) was calculated from [COD concentration in synthetic wastewater (mg-COD L^−1^)-COD concentration in broth (mg-COD L^−1^)]/COD concentration in synthetic wastewater (mg-COD L^−1^) × 100 (%). The average value of the total COD concentration in synthetic wastewater was 649 ± 44.7 mg L^−1^ (mean ± standard deviation). The COD removal efficiency was measured in the MFC pH 7.0, 5.5, and 4.0 fermentation broths throughout the operation. The concentrations of organic acids were determined using a high-performance liquid chromatograph system (Shimadzu, Kyoto, Japan) equipped with a refractive index detector (RID-10A, Shimadzu) and an organic acid analysis column (Aminex HPX-87H; Bio-Rad, Tokyo, Japan). Coulombic efficiency (*C.E.*) was calculated based on the COD removal (CODin–CODout) and the measured current using the values 1 g of COD = 0.125 mol of electrons and 1 A = 5.39 × 10^23^ electrons per day (Logan et al. 2006). Polarization and power density curves were plotted using a potentiostat (HA-1510; Hokuto Denko, Tokyo, Japan) (Shimoyama et al. 2008). Maximum power densities [*P*_*max*_ (mW m^−2^), peaks in the power density curves] and the short-circuit current density [*J*_*sc*_ (mA m^−2^)] were determined from these curves (Kato et al. 2010).

### Quantitative-PCR for *Geobacter* spp.

A DNA sample was extracted from the surface of the anode graphite felt piece (1 × 2 cm) after MFC operation using an ISOPLANT II kit (NIPPON GENE, Tokyo, Japan) according to the manufacturer’s instructions. Quantitative PCR assays were performed using a real-time PCR system (StepOnePlus; Applied Biosystems, Foster City, CA, USA) and SYBR Green Real-Time PCR Master Mix kit (Applied Biosystems), as reported previously (Kato et al. 2010). The abundances of total bacteria and family *Geobacteraceae* were separately quantified with the primer sets B1055F (5′-ATG GYT GTC GTC AGCT-3′) and B1392R (5′-ACG GGC GGT GTG TAC -3′) (Ritalahti et al. 2006), and Geo494F (5′-AGG AAG CAC CGG CTAACT CC-3′) and Geo825R (5′-TAC CCG CRA CAC CTA GT-3′) (Kato et al. 2010), respectively. Standard curves were generated using serially diluted genomic DNA from *G. sulfurreducens* (0.01–1 ng µL^−1^). At least two separate trials were conducted on each DNA sample.

### Clone library analysis

PCR amplification was performed using AmpliTaqGold (Applied Biosystems). The primer sets used were Ba27f (*Escherichia coli* positions 8–27) and Ba907r (*E. coli* positions 907–926) for the domain *Bacteria* (Lueders and Friedrich 2002). The PCR protocol entailed an initial denaturation for 10 min at 94 °C; 25 cycles of denaturation for 45 s at 94 °C, annealing for 30 s at 52 °C, and elongation for 90 s at 72 °C, followed by a final 5-min elongation at 72 °C. The PCR amplification products were purified using a QIAquick PCR purification kit (QIAGEN, Hilden, Germany), following which the ligated PCR products were transformed into *E. coli* JM109 by using a pGEM-T Easy Vector System (Promega, Fitchburg, WI, USA). The plasmids were extracted from the cloned cells and purified using a GenElute plasmid Miniprep Kit (Sigma-Aldrich, St. Louis, MO, USA), after which sequencing reactions were carried out with a BigDye Terminator v 3.1 Cycler Sequencing Kit (Applied Biosystems). Nucleotide sequencing was performed with an Applied Biosystems 3130*xl* Genetic Analyzer (Applied Biosystems). All sequences were checked for chimeric artifacts using the Bellerophon algorithm (http://comp-bio.anu.edu.au/bellerophon/bellerophon.pl). Sequences with ≥97.0% similarity were grouped into operational taxonomic units (OTUs). Nucleotide sequences were compared to the DDBJ/EMBL/GenBank databases using the BLAST algorithm (http://www.ncbi.nlm.nih.gov/blast/Blast.cgi). The nucleotide sequence data obtained in this study have been deposited in the DDBJ/EMBL/GenBank nucleotide sequence databases under the accession numbers LC168126 to LC168150.

### Metabolite extraction and quenching

Cells were collected from the anode graphite felt after 30 days’ operation at pH 7.0 or 4.0. The cell weight in each sample was adjusted to the same level based on optical density at 600 nm (OD_600_) before filtering each sample through a polytetrafluoroethylene membrane filter (Omnipore, 0.45 µM, 47-mm diameter; Millipore, Danvers, MA, USA). The dry cell weight of each sample was estimated by multiplying the determined cell weight of *E. coli* by the OD_600_ using the equation: dry cell weight (mg) = 0.0582 × OD_600_ × volume (mL). Immediately after filtration, the cells were washed in place with cold phosphate-buffered saline (137 mM NaCl, 8.10 mM Na_2_HPO_4_, 2.68 mM KCl, and 1.47 mM KH_2_PO_4_). The membrane filters with the washed cells were transferred to 50-mL centrifuge tubes and then frozen in liquid nitrogen. Metabolites were extracted from the cells using a modified cold chloroform-methanol method (Putri et al. 2013). Finally, the water phase of the extract (700 µL) was dried under vacuum and stored at −80 °C until use for mass analyses (Bennett et al. 2008).

### GC-Q-MS and LC-QqQ-MS analyses

The dried extract samples were thawed on ice, derivatized at 30 °C for 90 min with 100 µL of 20 mg mL^−1^ methoxyamine hydrochloride in pyridine, then 50 µL *N*-methyl-*N*-(trimethylsilyl) trifluoroacetamide (GL Sciences, Tokyo, Japan) (Lisec et al. 2006) was added followed by incubation at 37 °C for 30 min. Samples (1 µL) of the derivatives were subjected to gas chromatography-quadrupole-mass spectrometry (GC-Q-MS) analysis (GCMSQP-2010 system, Shimadzu) for detection of metabolites from the TCA cycle, as well as the detection of glutamate and glucose.

Aliquots of the dried extract samples were also dissolved in 50 µL Milli-Q water and prepared for analysis by liquid chromatography triple-stage quadrupole mass spectrometry (LC-QqQ-MS) (high-performance liquid chromatography: Agilent 1200 series, MS: Agilent 6460 with Jet Stream Technology, Agilent Technologies, Waldbronn, Germany) controlled by MassHunter Workstation Data Acquisition software (v. B. 04.01; Agilent Technologies). Metabolites from the EM and pentose phosphate (PP) pathways were detected, as well as of acetyl-CoA, adenosine triphosphate (ATP), adenosine diphosphate (ADP), nicotinamide adenine dinucleotide (NADH, NAD^+^), and nicotinamide adenine dinucleotide phosphate (NADPH, NADP^+^) (Luo et al. 2007). Details of the GC-Q-MS and LC-QqQ-MS operating conditions and methods have been described previously (Kato et al. 2012).

## Results

### Performance and electricity generation of the MFCs

**Fig. 2:**
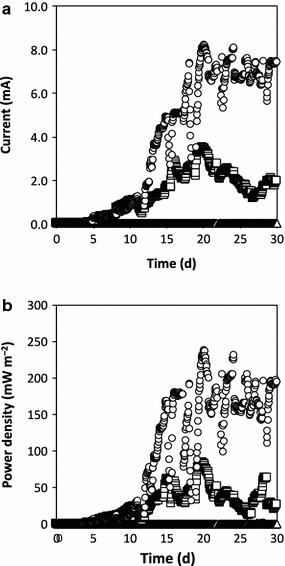
Average values of **a** current (mA) and **b** power density (mW m^−2^) were analyzed throughout the operation period (pH 7.0: *open circles*, pH 5.5: *open squares*, and pH 4.0: *open triangles*)

The physicochemical performances of the MFCs at pH 7.0, 5.5, and 4.0 were compared (Tables 1, 2; Fig. 2). Similar amounts of COD were degraded by the total microflora in each MFC (Table 1). However, the total concentration of organic acids was higher at day 30 in the MFCs operated at pH 5.5 and 4.0 compared to at pH 7.0, and the concentration of each organic acid measured was higher in the MFCs operated at pH 5.5 and 4.0 compared to at pH 7.0, with the concentration of acetate being the highest (Table 2). The accumulation of organic acids has often been observed in microflora cultures grown at low pH conditions (Sasaki et al. 2014). Differences in the environmental conditions between pH 7.0, 5.5, and 4.0 would affect the current-generating conditions in microflora on the surface of the anodic electrode.

**Table 1:** Biochemical and electrochemical performance during stable operation of microbial fuel cells (MFCs) operating at pH 7.0, 5.5, or 4.0

MFC	COD removal efficiency^a^ (%)	Total concentration of organic acids^b^ (mM)	*J*_*sc*_^c^ (mA m^−2^)	*P*_*max*_^d^ (mW m^−2^)	*C.E.* ^e^ (%)
pH 7.0	57.8 ± 8.82	0.47	410 ± 176	381 ± 155	34.0 ± 9.18
pH 5.5	57.1 ± 12.8	1.19	253 ± 52.1	147 ± 18.3	12.1 ± 3.11
pH 4.0	56.9 ± 3.61	1.80	40.7 ± 14.6	5.14 ± 1.64	1.06 ± 1.43

^a^Chemical oxygen demand (COD) removal efficiency (%) was calculated as follows: 100 × [COD concentration in synthetic wastewater (mg-COD L^−1^)–COD concentration in broth (mg-COD L^−1^)]/COD concentration in synthetic wastewater (mg-COD L^−1^)

^b^Sum of the organic acid concentrations in the suspended fractions of samples collected after 30 days’ operation at pH 7.0, pH 5.5, or pH 4.0

^c^Short-circuit current density [*J*_*sc*_ (mA m^−2^)] was determined from the power density curves

^d^Maximum power density [*P*_*max*_ (mW m^−2^)] was obtained from the peak in the power density curves

^e^Coulombic efficiency (*C.E.*) was calculated based on the COD removal (CODin–CODout) and the measured current, using 1 g of COD = 0.125 mol of electrons, and 1 A = 5.39 × 10^23^ electrons per day

**Table 2:** Organic acid concentrations in the liquid fractions of MFCs operated for 30 days at pH 7.0, pH 5.5, or pH 4.0

MFC	Organic acid concentration (mM)				
	Formate	Acetate	Propionate	Butyrate	Lactate
pH 7.0	ND	0.39	ND	0.08	ND
pH 5.5	0.27	0.44	0.19	0.30	ND
pH 4.0	0.18	0.62	0.47	0.47	0.06

*ND* not detected

Current production and power density in the MFC operated at pH 7.0 began to increase at day 5 and were stable from day 20 to day 30 (Fig. 2). The electrochemical performances of the pH 7.0, 5.5, and 4.0 reactors from day 20 to day 30 are summarized in Table 1. The values of *J*_*sc*_ (410 ± 176 mA m^−2^) and *P*_*max*_ (381 ± 155 mW m^−2^) for the MFC at pH 7.0 were similar to those previously reported for MFCs (Yoshizawa et al. 2014; Zhang et al. 2015). In contrast, little current production and low power density were observed in the MFCs operated at pH 5.5 and 4.0 throughout the operation. Accordingly, a much higher *C.E.* was observed in the MFC operating at pH 7.0 (neutral pH) than at pH 5.5 and 4.0 (acidic pH), in agreement with previous research by He et al. (2008). Because stable, middle, and low current production was verified for electrodes at pH 7.0, 5.5, and 4.0, respectively, genetic and metabolomic analyses were performed on microflora collected from the MFCs.

### Ratio of *Geobacter* species in the microflora of the MFCs

The relationship between current production and the ratio of *Geobacter* to the total bacterial cells was investigated. Quantitative analysis of total bacteria and family *Geobacteraceae* were conducted of the microflora collected from the anodic electrodes of MFCs operated at pH 7.0, 5.5, and 4.0 at day 30 (Table 3). Consistent with the observed current production, the amount and the ratio of family *Geobacteraceae* to the total cells on the electrode at pH 7.0 were higher than that at pH 5.5 and 4.0, and the *Geobacteraceae* ratio at pH 7.0 was similar to that reported in a previous study on electricity generation by microflora (Kouzuma et al. 2013).

**Table 3:** Quantitative PCR analysis of *Geobacteraceae* bacteria on the anodic electrode in MFCs at pH 7.0, pH 5.5, or pH 4.0

MFC	*Geobacteraceae* bacteria (ng cm^−2^)^a^	Ratio of *Geobacteraceae* (%)
pH 7.0	965 ± 7.50	26.7 ± 2.72
pH 5.5	34.6 ± 11.1	1.57 ± 0.51
pH 4.0	0.12 ± 0.02	0.10 ± 0.02

^a^The amount of DNA mass (ng) per anode graphite felt sample (cm^−2^)

Bacterial clone libraries of 16S rRNA genes were constructed to further elucidate the genetic sequences of microbes belonging to family *Geobacteraceae* and other microbes on the anodic electrodes (Retained) and in the fermentation broths (Suspended) sampled on day 30 from MFCs operated at pH 7.0 and 4.0 (Table 4; Additional file 1: Table S1, respectively). Sequences related to microbes belonging to family *Geobacteraceae* were only detected in the retained fraction of the sample obtained from the anodic electrode of the MFC operated at pH 7.0 (Table 4). Six sequences out of a total of 39 clones in the retained fraction showed 98.6% similarity to *Geobacter sulfurreducens* (NR_075009).

**Table 4:** Phylogenetic affiliations and number of bacterial clones obtained from retained and suspended fractions in the MFC operated for 30 days at pH 7.0

OTU^a^	No. of clones^b^		Phylogenetic group	The closest isolated strain (accession no., similarity)	The closest environmental clone (accession no., similarity)	Isolated source of environmental clones
	Retained	Suspended				
1	6		*Proteobacteria*; *Deltaproteobacteria*	*Geobacter sulfurreducens* (NR_075009, 98.6%)	Uncultured bacterium (GQ152932, 100%)	Mixed consortium in MFC
2	4		*Proteobacteria*; *Gammaproteobacteria*	*Citrobacter freundii* (AB548830, 100%)	Uncultured organism (HQ745055, 99.9%)	Gastrointestinal specimens
3	3	1	*Proteobacteria*; *Gammaproteobacteria*	*Raoultella ornithinolytica* (CP004142, 100%)	Uncultured bacterium (EF515418, 100%)	Upflow MFC anode
4	4	1	*Proteobacteria*; *Gammaproteobacteria*	*Pseudomonas fragi* (AB685617, 99.8%)	Uncultured bacterium (EU469649, 99.8%)	Mammalian gut microbes
5		5	*Proteobacteria*; *Gammaproteobacteria*	*Aeromonas dhakensis* (KF938660, 100%)	Uncultured bacterium (JX271966, 100%)	Activated sludge from large-scale
6		3	*Proteobacteria*; *Gammaproteobacteria*	*Aeromonas sharmana* (NR_043470, 96.9%)	Uncultured *Aeromonas* sp. (EF679186, 98.6%)	Sewage plant Anaerobic sludge from MFC
7	1		*Proteobacteria*; *Betaproteobacteria*	*Advenella kashmirensis* (KF956701, 95.1%)	Uncultured bacterium (FN563161, 99.7%)	Mesophilic biogas digester
8	1	1	*Proteobacteria*; *Betaproteobacteria*	*Azospira oryzae* (DQ863512, 94.1%)	Uncultured *Azospira* sp. (JF736645, 96.5%)	Biofilm on electrode in MFC
9	12	5	*Bacteroidetes*; *Cytophagia*	*Pontibacter korlensis* (GQ503321, 82.7%)	*Bacteriodetes* bacterium (JX828412, 95.9%)	Iron-reducing enrichment culture
10	3		*Bacteroidetes*; *Bacteroidia*	*Bacteroides nordii* (NR_112939, 92.9%)	Uncultured bacterium (KC179060, 99.7%)	Activated sludge
11		12	*Bacteroidetes*; *Bacteroidia*	*Dysgonomonas oryzarvi* (NR_113063, 99.7%)	Uncultured *Dysgonomonas* sp. (JX535216, 100%)	Activated sludge from sewage
12		1	*Bacteroidetes*; *Bacteroidia*	*Dysgonomonas capnocytophagoides* (NR_113133, 93.2%)	Uncultured bacterium (HQ728219, 96.1%)	Glucose-fed MFC
13	3		*Firmicutes*; *Negativicutes*	*Anaeroarcus burkinensis* (NR_025298, 97.7%)	Uncultured bacterium (AB329655, 99.9%)	UASB granule
14	2		*Firmicutes*; *Negativicutes*	*Acidaminococcus fermentans* (NR_074928, 93.8%)	Uncultured bacterium (JN680060, 99.5%)	Anode biofilm at psychrotolerants in MFC
15		1	*Firmicutes*; *Clostridia*	*Pseudoflavonifractor capillosus* (NR_025670, 89.8%)	Uncultured *Clostridiales* bacterium (FJ393118, 91.0%)	Glucose-fed MFC anode
Sum	39	30				

^a^Sequences with more than 97.0% homology were considered as being the same as the operational taxonomic unit (OTU)

^b^Suspended: the suspended fraction obtained from fermentation broth, Retained: the retained fraction obtained from anode graphite felts

### TCA cycle at pH 7.0, 5.5, and 4.0

**Fig. 3:**
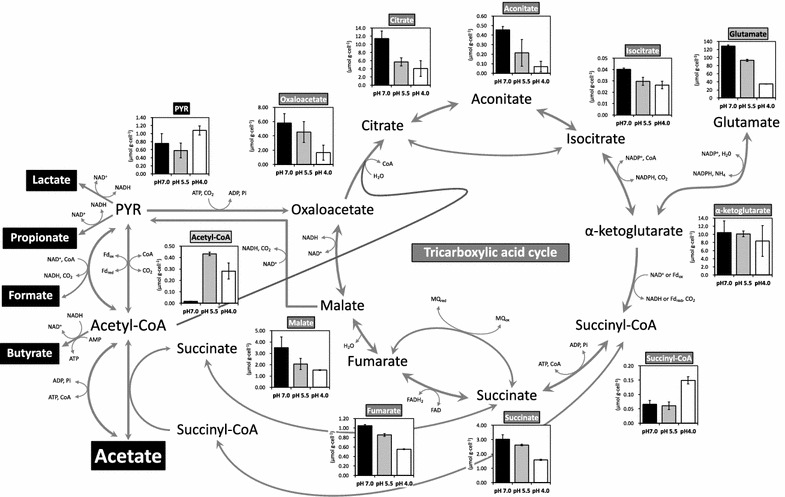
Comparison of intracellular metabolite concentrations related to the TCA cycle in microbial cells growing on the MFC anode (pH 7.0: *black bars*, pH 5.5: *gray bars*, and pH 4.0: *white bars*). The metabolite concentrations (µmol g-cell^−1^) were measured after 30 days’ operation. *Error bars* indicate ± standard deviation. The metabolic flow of organic acids (formate, acetate, propionate, butyrate and lactate) are shown. Acetyl-CoA and pyruvate relate to the metabolism of acetate by the TCA cycle in *G. sulfurreducens* (Mahadevan et al. 2006). Glutamate is one of the important metabolite in microbial nitrogen metabolism (Reitzer 2003). *AMP* adenosine-monophosphate, *CoA* coenzyme A, *FAD* flavin adenine dinucleotide, *PYR* pyruvate, *Fd* ferredoxin, *MQ* menaquinone, *Pi* phosphoric acid

Synthetic wastewater containing starch as the major carbon source was continuously flowed into the MFCs and the metabolic state related to the TCA cycle of the microflora on the anodic electrode was analyzed after operating for 30 days at pH 7.0, 5.5, or 4.0 (Fig. 3). The concentration of intracellular metabolites (µmol) was compared between pH 7.0, 5.5, and 4.0, based on the same cell weight (g-cell). The levels of most of the intracellular metabolites (oxaloacetate, citrate, aconitate, isocitrate, succinate, fumarate, and malate) related to the TCA cycle at pH 7.0, 5.5, and 4.0 were correlated with current densities (Fig. 3).

### Glycolysis (EM and PP pathways) at pH 7.0, 5.5, and 4.0

The profile of intercellular metabolites related to glycolysis (EM and PP pathways) differed in the microflora samples obtained from the anodes of reactors operated at pH 7.0, 5.5, and pH 4.0 (Additional file 1: Figure S1). Correlations were not observed between the levels of most metabolites related to glycolysis and current density. The intracellular concentrations of acetyl-CoA were higher at pH 4.0 and pH 5.5 than at pH 7.0, implying that the accumulation of organic acids at pH 4.0 and pH 5.5 inhibited carbon flux via acetyl-CoA (Fig. 3).

### Ratio of coenzymes at pH 7.0 and 4.0

**Fig. 4:**
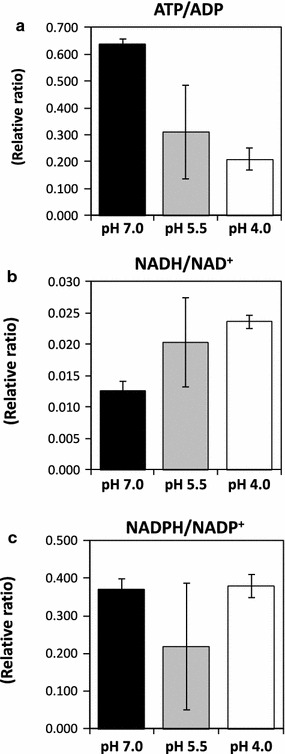
The relative ratios of **a** ATP/ADP, **b** NADH/NAD^+^, and **c** NADPH/NADP^+^ in the microbial fuel cells (pH 7.0: *black bars*, pH 5.5: *gray bars*, and pH 4.0: *white bars*) were calculated based on the amounts of these metabolites

A previous study of the metabolic state of *G. sulfurreducens* cells showed that the ratios of intracellular coenzyme concentrations (high ATP/ADP, low NADH/NAD^+^, and low NADPH/NADP^+^) correlated well with electricity generation (Song et al. 2016). As expected, the ATP/ADP ratio in microflora at pH 7.0 was larger than at pH 5.5 and 4.0 (Fig. 4). The NADH/NAD^+^ ratio at pH 7.0 was similar to that at pH 5.5 and was smaller than that at pH 4.0. On the other hand, the NADPH/NADP^+^ ratio at pH 7.0 was similar to that at pH 5.5 and 4.0.

## Discussion

The observed good current generation at neutral pH and poor current generation below pH 5.0 were in accordance with the previous study of Patil et al. (2011). In the present study, microorganisms related to *Geobacter* species would play a major role in current generation at the anode electrode (Lovley 2008). *Geobacter* cells oxidize acetate via the TCA cycle to generate electricity (Mahadevan et al. 2006). Good correlation between the levels of metabolites relating to the TCA cycle and current generation is corroborated by our previous study showing that the intracellular metabolites in the TCA cycle are present at high concentration and activation of the TCA cycle supports higher electricity generation by a pure *Geobacter* culture (Song et al. 2016). The observed decrease in *Geobacter* cells at lower pH values and current densities indicates that the metabolic state of the TCA cycle of microflora directly reflects the state of the *Geobacter* cells, even though the biofilm on the surface of the anode included microorganisms other than *Geobacter*. Thus, the present results indicate that acetate produced from starch-derived glucose through acetyl-CoA is consumed via the TCA cycle in microflora, comprised mainly of *Geobacter* cells, for electricity generation at pH 7.0 (Fig. 3).

In addition, a higher intracellular concentration of glutamate was observed at pH 7.0 (Fig. 3). This correlates to higher cell numbers on the electrode at pH 7.0 compared to at pH 5.5 and 4.0 (data not shown) and supports the central importance of glutamate at the intersection between carbon and nitrogen metabolism required for bacterial growth (Commichau et al. 2008).

The profile of intercellular metabolites related to glycolysis (EM and PP pathways) at pH 7.0, 5.5, and pH 4.0 showed no relationship with current density (Additional file 1: Figure S1). *Geobacter* cells reportedly down-regulate gluconeogenesis during electricity generation (Meng et al. 2013; Song et al. 2016). Because *G. sulfurreducens* cells cannot directly utilize glucose (Mahadevan et al. 2006), we can separate the phases of current production by microflora into an acetate-producing phase from starch via glycolysis, and a current-producing phase from acetate via the TCA cycle. Thus, intracellular metabolites related to glycolysis would be affected by carbon flux from starch-derived glucose in microorganisms other than *Geobacter* cells. In contrast, the intracellular concentrations of acetyl-CoA were higher at pH 5.5 and 4.0 than at pH 7.0, implying that the accumulation of organic acids at pH 5.5 and 4.0 inhibited carbon flux via acetyl-CoA (Fig. 3). The same profiles were observed in microflora used for methane fermentation from glucose (Sasaki et al. 2014).

ATP is produced in the respiratory electron transfer chain and oxidative consumption of NADH occurs when electricity generation is high in *Geobacter* species (Meng et al. 2013). ATP is also produced in the EM pathway by substrate level phosphorylation. Thus, increased ATP/ADP would be due to activation of the EM pathway (by consumption of starch-derived glucose) and TCA cycle (by electricity generation) at pH 7.0 (Fig. 4). In addition, the observed decrease in the NADH/NAD^+^ ratio of the microflora at pH 7.0 was as expected, whereas the reason for the fluctuating NADH/NAD^+^ ratio at pH 5.5 remains unclear. In contrast, the NADPH/NADP^+^ ratio in the microflora was the same at pH 7.0, 5.5, and 4.0, although oxidative consumption of NADPH is faster in electricity-generating *Geobacter* cells (Song et al. 2016). During electricity generation, NADPH is produced in the TCA cycle (isocitrate to α-ketoglutarate) and consumed during glutamate production and in the respiratory chain. In this study, NADPH was additionally supplied from the PP pathway, resulting in an increased NADPH/NADP^+^ ratio at pH 7.0 and 5.5.

In this study, metabolomic analyses of the microflora on the anodic electrode of a MFC showed for the first time that activation of the TCA cycle and increased ATP generation are necessary for efficient current generation from starch-like waste, as observed in single kind of microorganism, *Geobacter* cells.

## Additional file

**Additional file 1.** **Table S1.** Phylogenetic affiliations and number of bacterial clones obtained from retained and suspended fractions in MFC operated for 30 days at pH 4.0. **Figure S1.** Comparison of intracellular metabolite concentrations related to central metabolism in microbial cells growing on the MFC anode at pH 7.0, pH 5.5, and pH 4.0.

## Abbreviations

MFCs: microbial fuel cells
TCA: tricarboxylic acid
ATP: adenosine triphosphate
AMP: adenosine diphosphate
EM: Embden-Meyerhof
PTFE: 4-polytetrafluoroethylene
DSMZ: Deutsche Sammlung von Mikroorganismen und Zellkulturen
*R*_*ext*_: initial external resistance
COD: chemical oxygen demand
*C.E.*: coulombic efficiency
*P*_*max*_: maximum power density
*J*_*sc*_: short-circuit current density
DNA: deoxyribonucleic acid
PCR: polymerase chain reaction
OTUs: operational taxonomic units
OD_600_: optical density at 600 nm
GC-Q-MS: gas chromatography-quadrupole-mass spectrometry
LC-QqQ-MS: liquid chromatography triple-stage quadrupole mass spectrometry
PP: pentose phosphate
NADH: nicotinamide adenine dinucleotide
NADPH: nicotinamide adenine dinucleotide phosphate
1,3 BPG: 1,3-bisphosphoglycerate
2PG: 2-phosphoglycerate
3PG: 3-phosphoglycerate
6PGA: 6-phosphogluconate
6PGL: 6-phosphogluconolactone
AMP: adenosine-monophosphate
CoA: coenzyme A
DHAP: dihydroxyacetone phosphate
E4P: erythrose-4-phosphate
F6P: fructose-6-phosphate
FAD: flavin adenine dinucleotide
FBP: fructose-1,6-bisphosphate
Fd: ferredoxin
G6P: glucose-6-phosphate
GAP: glyceraldehyde-3-phosphate
MQ: menaquinone
PEP: phosphoenolpyruvate
Pi: phosphoric acid
PPi: pyrophosphoric acid
PYR: pyruvate
R5P: ribose-5-phosphate
Ru5P: ribulose-5-phosphate
S7P: sedoheptulose-7-phosphate
X5P: xylulose-5-phosphate

## References

[CR1] Bennett BD, Yuan J, Kimball EH, Rabinowitz JD (2008). Absolute quantitation of intracellular metabolite concentrations by an isotope ratio-based approach. Nat Protoc.

[CR2] Commichau FM, Gunka K, Landmann JJ, Stülke J (2008). Glutamate metabolism in *Bacillus subtilis*: gene expression and enzyme activities evolved to avoid futile cycles and to allow rapid responses to perturbations of the system. J Bacteriol.

[CR3] Guerrero L, Montalvo S, Coronado E, Chamy R, Poirrier P, Crutchik D, Sánchez E, De La Rubia MA, Borja R (2009). Performance evaluation of a two-phase anaerobic digestion process of synthetic domestic wastewater at ambient temperature. J Environ Sci Health A Tox Hazard Subst Environ Eng.

[CR4] He Z, Huang Y, Manohar AK, Mansfeld F (2008). Effect of electrolyte pH on the rate of the anodic and cathodic reactions in an air-cathode microbial fuel cell. Bioelectrochem.

[CR5] Kato S, Nakamura R, Kai F, Watanabe K, Hashimoto K (2010). Respiratory interactions of soil bacteria with (semi)conductive iron-oxide minerals. Environ Microbiol.

[CR6] Kato H, Izumi Y, Hasunuma T, Matsuda F, Kondo A (2012). Widely targeted metabolic profiling analysis of yeast central metabolites. J Biosci Bioeng.

[CR7] Kouzuma A, Kasai T, Nakagawa G, Yamamuro A, Abe T, Watanabe K (2013). Comparative metagenomics of anode-associated microbiomes developed in rice paddy-field microbial fuel cells. PLoS ONE.

[CR8] Lisec J, Schauer N, Kopka J, Willmitzer L, Fernie AR (2006). Gas chromatography mass spectrometry-based metabolite profiling in plants. Nat Protoc.

[CR9] Logan BE (2009). Exoelectrogenic bacteria that power microbial fuel cells. Nat Rev Microbiol.

[CR10] Logan BE, Hamelers B, Rozendal R, Schröder U, Keller J, Freguia S, Aelterman P, Verstraete W, Rabaey K (2006). Microbial fuel cells: methodology and technology. Environ Sci Technol.

[CR11] Lovley DR (2008). The microbe electric: conversion of organic matter to electricity. Curr Opin Biotechnol.

[CR12] Lovley DR (2012). Electromicrobiology. Annu Rev Microbiol.

[CR13] Lueders T, Friedrich MW (2002). Effects of amendment with ferrihydrite and gypsum on the structure and activity of methanogenic populations in rice field soil. Appl Environ Microbiol.

[CR14] Luo B, Groenke K, Takors R, Wandrey C, Oldiges M (2007). Simultaneous determination of multiple intracellular metabolites in glycolysis, pentose phosphate pathway and tricarboxylic acid cycle by liquid chromatography-mass spectrometry. J Chromatogr A.

[CR15] Mahadevan R, Bond DR, Butler JE, Esteve-Nuñez A, Coppi MV, Palsson BO, Schilling CH, Lovley DR (2006). Characterization of metabolism in the Fe(III)-reducing organism *Geobacter sulfurreducens* by constraint-based modeling. Appl Environ Microbiol.

[CR16] Meng J, Xu Z, Guo J, Yue Y, Sun X (2013). Analysis of enhanced current-generating mechanism of *Geobacter sulfurreducens* strain via model-driven metabolism simulation. PLoS ONE.

[CR17] Miyahara M, Hashimoto K, Watanabe K (2013). Use of cassette-electrode microbial fuel cell for wastewater treatment. J Biosci Bioeng.

[CR18] Patil SA, Harnisch F, Koch C, Hübschmann T, Fetzer I, Carmona-Martínez AA, Müller S, Schröder U (2011). Electroactive mixed culture derived biofilms in microbial bioelectrochemical systems: the role of pH on biofilm formation, performance and composition. Bioresour Technol.

[CR19] Putri SP, Nakayama Y, Matsuda F, Uchikata T, Kobayashi S, Matsubara A, Fukusaki E (2013). Current metabolomics: practical applications. J Biosci Bioeng.

[CR20] Reitzer L (2003). Nitrogen assimilation and global regulation in *Escherichia coli*. Annu Rev Microbiol.

[CR21] Ritalahti KM, Amos BK, Sung Y, Wu Q, Koenigsberg SS, Löffler FE (2006). Quantitative PCR targeting 16S rRNA and reductive dehalogenase genes simultaneously monitors multiple Dehalococcoides strains. Appl Environ Microbiol.

[CR22] Sasaki D, Sasaki K, Tsuge Y, Morita M, Kondo A (2014). Comparison of metabolomic profiles of microbial communities between stable and deteriorated methanogenic processes. Bioresour Technol.

[CR23] Shimoyama T, Komukai S, Yamazawa A, Ueno Y, Logan BE, Watanabe K (2008). Electricity generation from model organic wastewater in a cassette-electrode microbial fuel cell. Appl Microbiol Biotechnol.

[CR24] Song J, Sasaki D, Sasaki K, Kato S, Kondo A, Hashimoto K, Nakanishi S (2016). Comprehensive metabolomic analyses of anode-respiring *Geobacter sulfurreducens* cells: the impact of anode-respiration activity on intracellular metabolite levels. Process Biochem.

[CR25] Toya Y, Shimizu H (2013). Flux analysis and metabolomics for systematic metabolic engineering of microorganisms. Biotechnol Adv.

[CR26] Yoshizawa T, Miyahara M, Kouzuma A, Watanabe K (2014). Conversion of activated-sludge reactors to microbial fuel cells for wastewater treatment coupled to electricity generation. J Biosci Bioeng.

[CR27] Zhang L, Zhu X, Kashima H, Li J, Ye D, Liao Q, Regan JM (2015). Bioresource technology anolyte recirculation effects in buffered and unbuffered single-chamber air-cathode microbial fuel cells. Bioresour Technol.

