# Supplementary material for: Comparative metabolic state of microflora on the surface of the anode electrode in a microbial fuel cell operated at different pH conditions
Source: AMB Express. 2016 Dec 20;6:125. doi: 10.1186/s13568-016-0299-4 (PMC5174012; doi:10.1186/s13568-016-0299-4)

Additional File -1

Comparative metabolic state of microflora on the surface of the anode electrode in a microbial fuel cell operated at different pH conditions

AMB Express

Daisuke Sasaki[1], Kengo Sasaki[1], Yota Tsuge[2], and Akihiko Kondo[1,3,4]

[1] Graduate School of Science, Technology and Innovation, Kobe University, 1-1 Rokkodaicho, Nada-ku, Kobe, Hyogo 657-8501, Japan

[2] Institute for Frontier Science Initiative, Kanazawa University, Kakuma-machi, Kanazawa, Ishikawa 920-1192, Japan

[3] Graduate School of Engineering, Kobe University, 1-1 Rokkodai-cho, Nada-ku, Kobe, Hyogo 657-8501, Japan

[4] RIKEN Center for Sustainable Resource Science, 1-7-22 Suehiro-cho, Tsurumi-ku, Yokohama, Kanagawa 230-0045, Japan

Corresponding author: Prof. Akihiko Kondo (E-mail: akondo@kobe-u.ac.jp)

Table S1.

Phylogenetic affiliations and number of bacterial clones obtained from retained and suspended fractions in MFC operated for 30 days at pH 4.0.

OTU[a]	No. of clones[b]		Phylogenetic group	The closest isolated strain (accession no., similarity)	The closest environmental clone (accession no., similarity)	Isolated source of environmental clones
	Retained	Suspended				
**1**	**1**		*Proteobacteria*; *Deltaproteobacteria*	*Desulfovibrio desulfuricans* (NR_104990, 99.6%)	uncultured *Desulfovibrio* sp. (FJ393090, 99.7%)	MFC using glucose and lactate
**2**	**3**		*Proteobacteria*; *Gammaproteobacteria*	*Frateuria* sp. EC-K130 (AB264175, 100%)	uncultured bacterium (EF201736, 99.4%)	lake sediment core
**3**		**6**	*Proteobacteria*; *Gammaproteobacteria*	*Enterobacter asburiae* (JF772078, 99.7%)	uncultured *Citrobacter* sp. (GQ416219, 99.9%)	biological degreasing systems
**4**	**6**		*Proteobacteria*; *Betaproteobacteria*	*Pandoraea sputorum* (JQ839144, 100%)	*Pandoraea* sp. G5084 (AF247693, 100%)	blood cultures
**5**	**6**		*Proteobacteria*; *Betaproteobacteria*	*Burkholderia pseudomultivorans* (NR_117661, 98.6%)	*Burkholderia* sp. MAC25 (HM587748, 98.6%)	forest soil
**6**	**18**	**20**	*Bacteroidetes*; *Bacteroidia*	*Bacteroides stercoris* (NR_112943, 86.8%)	uncultured bacterium (EF515298, 99.4%)	upflow MFC anode
**7**		**10**	*Bacteroidetes*; *Bacteroidia*	*Prevotella paludivivens* (AB081578, 97.5%)	uncultured bacterium (JN245858, 99.9%)	wastewater treatment plant
**8**	**3**		*Firmicutes*; *Negativicutes*	*Pectinatus frisingensis* (NR_117702, 99.9%)	*Pectinatus* sp. H1 (FJ668028, 99.9%)	UASB hydrogen-producing bioreactor
**9**		**1**	*Firmicutes*; *Negativicutes*	*Selenomonas bovis* (JN613164, 90.7%)	uncultured bacterium (EU828397, 92.0%)	Hydrogen-producing bioreactor
**10**	**2**		*Firmicutes*; *Clostridia*	*Clostridium swellfunianum* (NR_126179, 98.4%)	uncultured bacterium (HQ266807, 98.8%)	rice field soil
**Sum**	**39**	**38**				

[a] Sequences with more than 97.0% homology were considered as being the same as the operational taxonomic unit (OTU).
[b] Suspended: the suspended fraction obtained from fermentation broth, Retained: the retained fraction obtained from anode graphite felts.

Figure S1.

Comparison of intracellular metabolite concentrations related to central metabolism in microbial cells growing on the MFC anode (pH 7.0: black bars, pH 5.5: gray bars, and pH 4.0: white bars). The metabolite concentrations (μmol g-cell^{-1}) were measured after 30 days' operation. Error bars indicate ± standard deviation.

Abbreviations: 1,3 BPG, 1,3-bisphosphoglycerate; 2PG, 2-phosphoglycerate; 3PG, 3-phosphoglycerate; 6PGA, 6-phosphogluconate; 6PGL, 6-phosphogluconolactone; AMP, adenosine-monophosphate; DHAP, dihydroxyacetone phosphate; E4P, erythrose-4-phosphate; F6P, fructose-6-phosphate; FBP, fructose-1,6-bisphosphate; G6P, glucose-6-phosphate; GAP, glyceraldehyde-3-phosphate; PEP, phosphoenolpyruvate; PYR, pyruvate; Pi, phosphoric acid; PPi, pyrophosphoric acid; R5P, ribose-5-phosphate; Ru5P, ribulose-5-phosphate; S7P, sedoheptulose-7-phosphate; X5P, xylulose-5-phosphate.

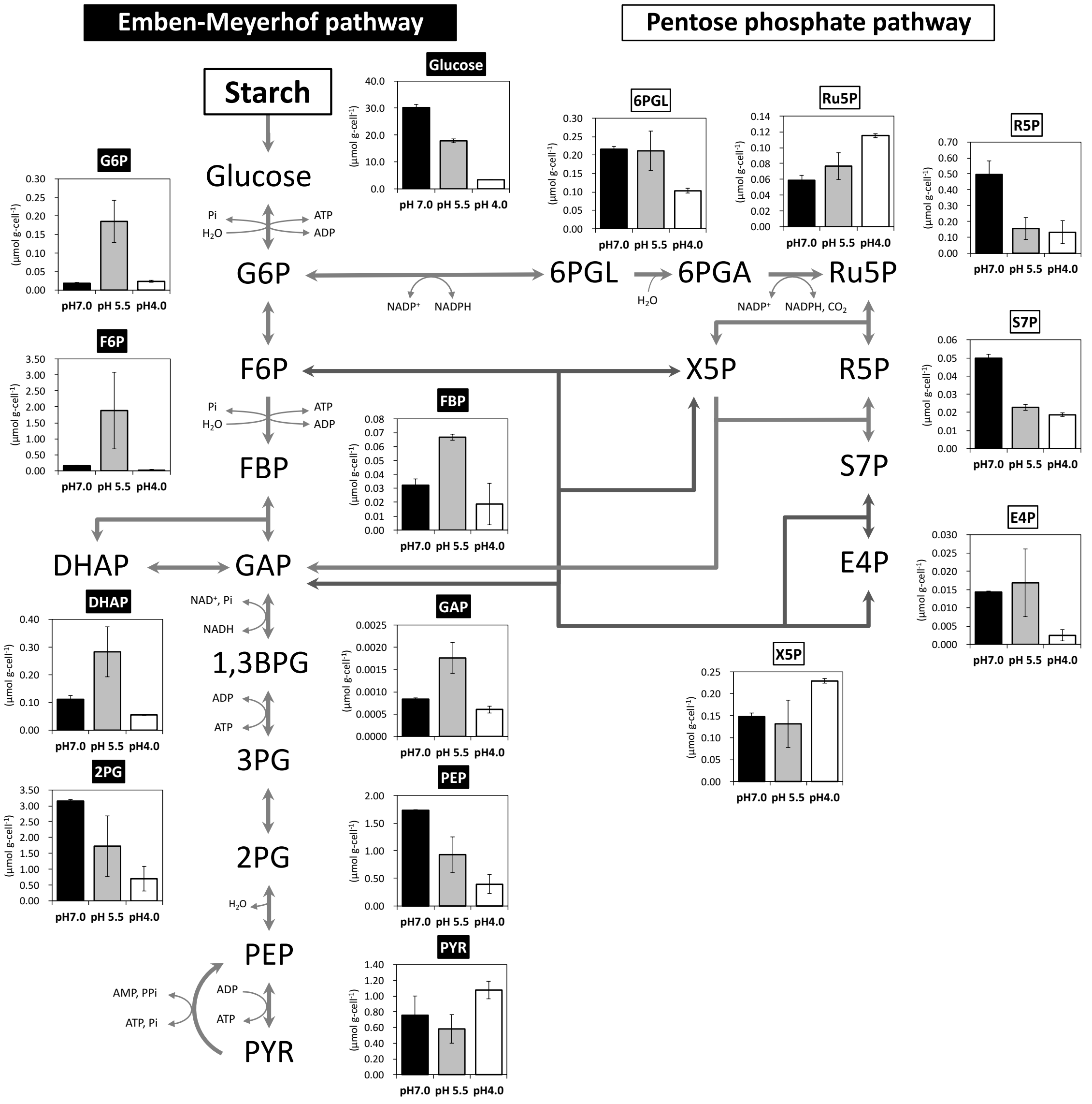

Supplement: Supplementary file 1 — Additional file 1. Table S1. Phylogenetic affiliations and number of bacterial clones obtained from retained and suspended fractions in MFC operated for 30 days at pH 4.0. Figure S1. Comparison of intracellular metabolite concentrations related to central metabolism in microbial cells growing on the MFC anode at pH 7.0, pH 5.5, and pH 4.0. [file 13568_2016_299_MOESM1_ESM.pdf]
